# Engineering Lineage Potency and Plasticity of Stem Cells using Epigenetic Molecules

**DOI:** 10.1038/s41598-018-34511-7

**Published:** 2018-11-02

**Authors:** Anandika Dhaliwal, Sandra Pelka, David S. Gray, Prabhas V. Moghe

**Affiliations:** 1 Department of Biomedical Engineering, Rutgers University, Piscataway, NJ United States; 2 Department of Chemical and Biochemical Engineering, Rutgers University, Piscataway, NJ United States

## Abstract

Stem cells are considered as a multipotent regenerative source for diseased and dysfunctional tissues. Despite the promise of stem cells, the inherent capacity of stem cells to convert to tissue-specific lineages can present a major challenge to the use of stem cells for regenerative medicine. We hypothesized that epigenetic regulating molecules can modulate the stem cell’s developmental program, and thus potentially overcome the limited lineage differentiation that human stem cells exhibit based on the source and processing of stem cells. In this study, we screened a library of 84 small molecule pharmacological agents indicated in nucleosomal modification and identified a sub-set of specific molecules that influenced osteogenesis in human mesenchymal stem cells (hMSCs) while maintaining cell viability *in-vitro*. Pre-treatment with five candidate hits, *Gemcitabine, Decitabine, I-CBP112, Chidamide*, and *SIRT1/*2 *inhibitor IV*, maximally enhanced osteogenesis *in-vitro*. In contrast, five distinct molecules, *4-Iodo-SAHA, Scriptaid, AGK2, CI-amidine* and *Delphidine Chloride* maximally inhibited osteogenesis. We then tested the role of these molecules on hMSCs derived from aged human donors and report that small epigenetic molecules, namely *Gemcitabine and Chidamide*, can significantly promote osteogenic differentiation by 5.9- and 2.3-fold, respectively. Taken together, this study demonstrates new applications of identified small molecule drugs for sensitively regulating the lineage plasticity fates of bone-marrow derived mesenchymal stem cells through modulating the epigenetic profile of the cells.

## Introduction

There is a consensus in the scientific community about the potential of adult stem cell-based therapies and tissue regeneration^1–3^. Varied biochemical and biophysical cues have been employed to design efficacious cell-based therapies^3–5^. However, the ability to optimally harness the potential of adult human mesenchymal stem cells (hMSCs) and direct optimal cell phenotypic development for tissue formation continues to present a major challenge, which limits the translation of these technologies to the clinic. A major limitation is that the differentiation capabilities of MSCs deteriorate with age^6,7^ and with *in-vitro* passages^8^, thereby affecting their developmental potential and impairing the efficacy of cell therapy. The second major limitation is the poor stability of cell phenotypes^9^, which complicates the ability to accurately postulate the response of cells to engineered cues. Therefore, technologies that can enhance the potency of stem cells cultured *in-vitro* and modulate their sensitivity and stability to engineered cues, need to be developed to ensure a specific developmental fate of the cell and facilitate the advancement of cell-based therapies for tissue engineering applications.

Conventional regenerative tissue technologies have relied on extracellular signals (growth factors, small molecules and metabolic regulators) to accelerate lineage conversion and ameliorate age related MSC dysfunction^10–12^. While recent scientific evidence indicated that the epigenetic profile of the cell is a key determinant in guiding the developmental pathway of cells^13,14^, the role of epigenetic modifications in steering cell differentiation and the use of pharmacologic agents as epigenetic manipulators to optimize specific cell phenotypic development has not been explored. “Epigenetics” refers to the “non-genetically based cellular memory”, which involves heritable changes in gene expression that occur without alteration in DNA sequence. These changes can be a consequence of environmental factors or induced spontaneously, using two primary mechanisms of DNA methylation and covalent modification of histones^15^. The emerging field of epigenetics has thus far caught the interest of scientists globally by evidencing that the epigenetic markers influence gene expression and genome function, thereby directing DNA-based biological processes^15,16^. Recent studies have indicated the potential role of epigenetic modifiers such as trichostatin A, valproic acid and sodium butyrate in osteogenic differentiation^17–19^. Even so, the use of the many accessible pharmacologic agents as epigenetic manipulators and their application in optimizing specific cell phenotypic development has not been comprehensively realized.

**Figure 1:**
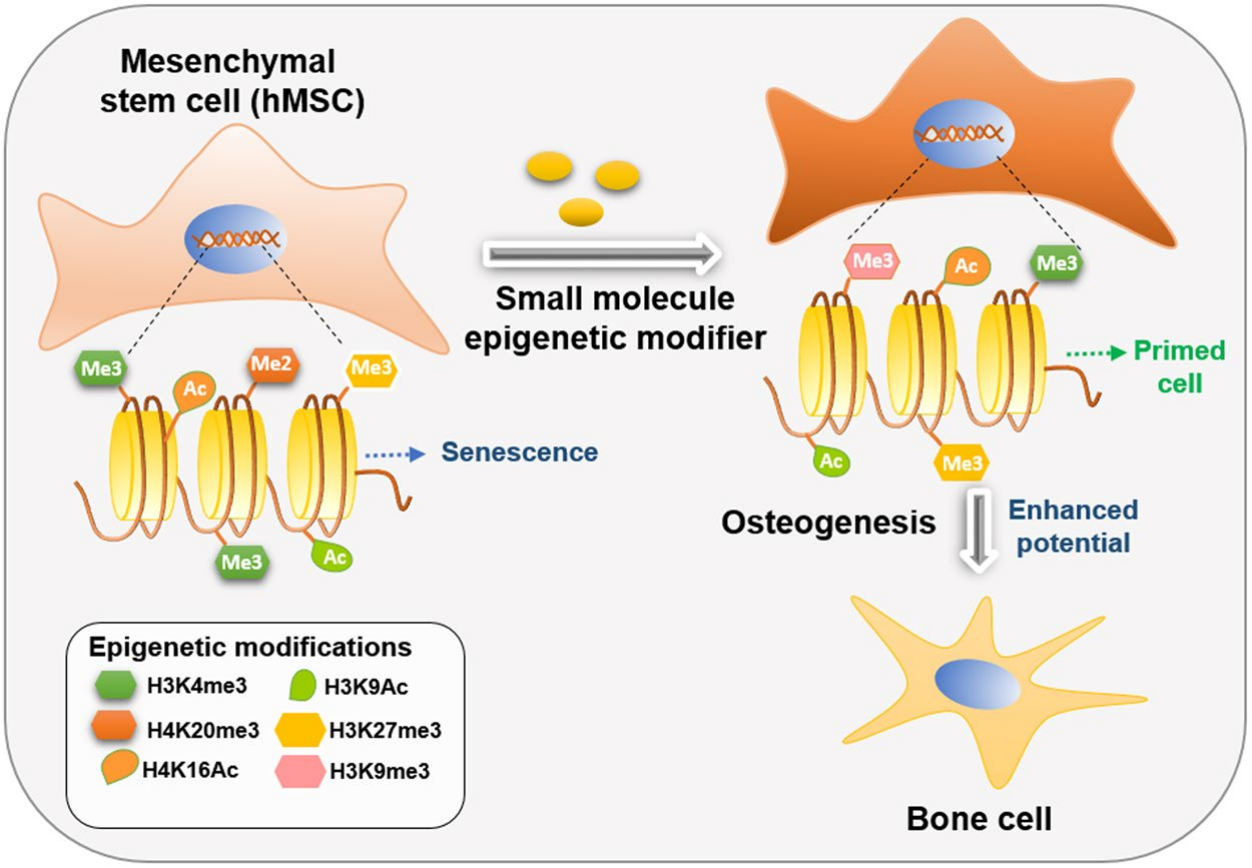
Increasing differentiation potential of *ex-vivo* cultured stem cells through epigenetic modulation. In this study small molecules nucleosomal modifiers able to significantly increase osteogenic differentiation potential of hMSCs were identified.

In this study, we systematically evaluated a library of pharmacological agents indicated in nucleosomal modification to identify specific compounds capable of modulating osteogenic differentiation (Fig. 1). 84 compounds capable of influencing the epigenetic profile of the cells and consequently the nucleosomal organization were screened (Table 1). The compounds included small molecules that modulate the activity of methyltransferases, demethylases, HATs, HDACs and acetylated lysine reader proteins. Top 10 compounds maximally enhancing or inhibiting osteogenesis in human mesenchymal stem cells (hMSCs) cultured *in vitro*, while maintaining cell viability, were identified and presented.

**Table 1:** List of all nucleosomal modifying drugs screened for modulating hMSC differentiation.

List of drugs screened		
(1) AGK2	(29) Delphinidin chloride	(57) Oxamflatin
(2) CAY10433	(30) ITF 2357	(58) 2′,3′,5′-triacetyl-5-Azacytidine
(3) M 344	(31) 3-Deazaneplanocin A	(59) Salermide
(4) HNHA	(32) Suramin (sodium salt)	(60) Mirin
(5) Octyl-α-ketoglutarate	(33) PFI-1	(61) UNC1999
(6) Cl-Amidine (trifluoroacetate salt)	(34) 5-Azacytidine	(62) Pimelic Diphenylamide 106
(7) CAY10669	(35) Chaetocin	(63) MS-275
(8) HC Toxin	(36) Decitabine	(64) RG-108
(9) JGB1741	(37) (+)-JQ1	(65) S-Adenosylhomocysteine
(10) Garcinol	(38) (—)-JQ1	(66) UNC0224
(11) MI-2 (hydrochloride)	(39) BSI-201	(67) Chidamide
(12) Sinefungin	(40) IOX1	(68) Pyroxamide
(13) Suberohydroxamic Acid	(41) MI-nc (hydrochloride)	(69) N-Oxalylglycine
(14) Valproic Acid	(42) Gemcitabine	(70) WDR5-0103
(15) Resveratol	(43) Lomeguatrib	(71) AMI-1 (sodium salt)
(16) 3-amino Benzamide	(44) Daminozide	(72) UNC1215
(17) 4-iodo-SAHA	(45) GSK-J1 (sodium salt)	(73) GSK 343
(18) C646	(46) GSKJ2 (sodium salt)	(74) Bromosporine
(19) Ellagic Acid	(47) GSK-J4 (hydrochloride)	(75) SIRT1/2 Inhibitor IV
20) Scriptaid	48) GSK-J5 (hydrochloride)	76) I-CBP112 (hydrochloride)
(21) UNC0321 (trifluoroacetate salt)	(49) Tenovin-1	(77) PFI-3
(22) (-)-Neplanocin A	(50) Tenovin-6	(78) 2,4-DPD
(23) F-Amidine (trifluoroacetate salt)	(51) BIX01294 (hydrochloride hydrate)	(79) DMOG
(24) UNC0638	(52) Anacardic Acid	(80) Trichostatin A
(25) Phthalazinone pyrazole	(53) CAY10603	(81) CAY10398
(26) Isoliquiritigenin	(54) Splitomicin	(82) RSC-133
(27) CCG-100602	(55) CBHA	(83) Piceatannol
(28) Zebularine	(56) Oxamflatin	(84) CAY10591

In parallel, we also elucidated the cross-talk between the epigenetic effects and stem cell lineage phenotypes using single cell nucleosomal imaging and image informatics. To accomplish this, we performed high content image informatics to track and annotate cells using SC-35 as a surrogate marker^20^, which can be employed to profile changes the *in-situ* nucleosomal organization globally after exposure to small molecule modifiers.

SC-35 nuclear speckle domains constitute small nuclear ribonucleoprotein particles (snRNPs), spliceosomes, and transcription factors that mediate co-transcriptional modifications of RNA^21,22^. Recent body of work from our lab has shown that speckle factor SC-35 can be employed as an integrative surrogate marker to assess the effect of environmental factors (growth factors, topography, biomaterials) on MSC differentiation and parse the emergent hMSC phenotypes predictably within 72 hours of exposure to external modulating factors^20,23^. We believe that treatment with these small molecules modifies the epigenetic profile, which in turn influences the regulation of gene expression and consequently the SC-35 spatial organization. SC-35 can therefore be utilized as a universal surrogate marker to annotate the cells by mapping the resultant textural signatures, capturing minute variations in nucleosomal organization, post treatment with epigenetic manipulators. Therefore, this is the first study to demonstrate that osteogenic differentiation can be regulated through epigenetic modulation by small molecules (Fig. 1), and that high content image informatic of SC-35 spatial organization can be employed to parse the resultant variances in nucleosomal organization.

## Results

### Optimization of osteogenic differentiation by modulating nucleosomal organization through small molecule pharmacologic agents

**Figure 2:**
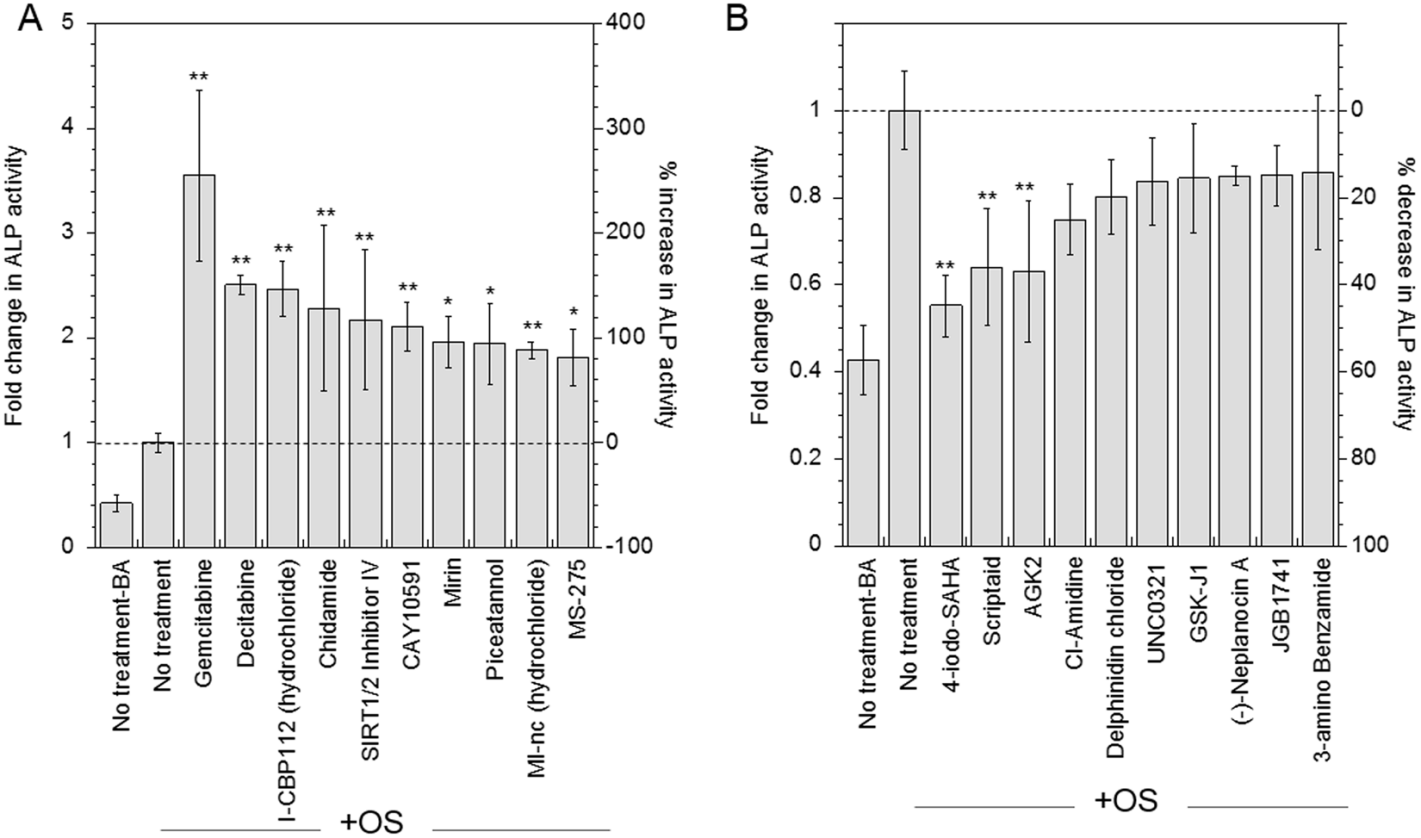
Small molecule nucleosomal modifiers influence osteogenic differentiation of hMSCs. The effect of treatment with pharmacological agents influencing the epigenetic profile of the cell on osteogenic differentiation was analyzed at Day 14 using ALP activity assay. Top 10 agents that significantly increased the ALP activity (**A**) and decreased ALP activity (**B**) were identified through screening. Statistical analysis was performed using the Dunnett Multiple Comparisons test, which compares all columns versus a control column (OS-no treatment). The symbols ** and * represent a significant change in ALP activity with respect to OS – no treatment condition to the level of p < 0.01 and p < 0.05, respectively.

A screen of 84 small molecule drugs known to influence nucleosomal organization (Table 1) was applied to identify the drugs that significantly influence osteogenic differentiation of cultured hMSCs *in vitro*. Cells were pretreated with drugs for 24 hours and subsequently induced to differentiate using commonly used cocktail of differentiation factors (osteogenic medium). The effect of drug treatment on osteogenesis was assessed at 14 days using ALP assay (Supplemental Fig. 1). The top 10 drugs that most increased osteogenesis or decreased osteogenesis were identified (Fig. 2A,B). Their concentrations and chemical structures are listed in Supplemental Tables 1,2. The top 5 drugs that significantly increased (p < 0.05) osteogenesis included *Gemcitabine, Decitabine, I-CBP112, Chidamide*, and *SIRT1/2* inhibitor IV. *Gemcitabine* maximally increased ALP activity by 3.5-fold, *Decitabine* and *I-CBP112* increased ALP activity by 2.5-fold, and *Chidamide* and *SIRT1/2 Inhibitor IV* increased ALP activity by 2.3- and 2.2-fold, respectively (Fig. 2A).

The top 5 drugs that most decreased osteogenesis were *4-Iodo SAHA, Scriptaid, AGK2, CI-Amidine* and *Delphidine Chloride*. Specifically, *4-Iodo-SAHA* significantly inhibited ALP activity by 45%, followed by *Scriptaid* and *AGK2*, which inhibited ALP activity by at least 36% (Fig. 2B). Furthermore, there was no significant difference in ALP activity between cells cultured in basal media, and cells cultured in osteogenic media after pretreatment with *4-Iodo-SAHA, Scriptaid* or *AGK2*, indicating that nucleosomal modifications by these drugs inhibit osteogenic differentiation completely even after induction with soluble factors.

The cells were also stained for ALP using Fast Blue RR salt staining (Supplemental Fig. 4), osteogenic differentiation marker RUNX2 using immune-staining and actin using phalloidin staining (Supplemental Fig. 5). The top 5 drugs identified to increase osteogenesis have more Fast Blue positive staining and RUNX2 expression, as compared to basal condition (BA), and to a level same or more than untreated cells cultured in osteogenic media (OS). Also, as observed from Supplemental Fig. 5, cells pre-treated with drugs identified to increase osteogenic differentiation, led to confluent mono-layers at 14-days, similar in morphology to untreated cells.

### Effect of small molecule pharmacologic agents on cell viability

**Figure 3:**
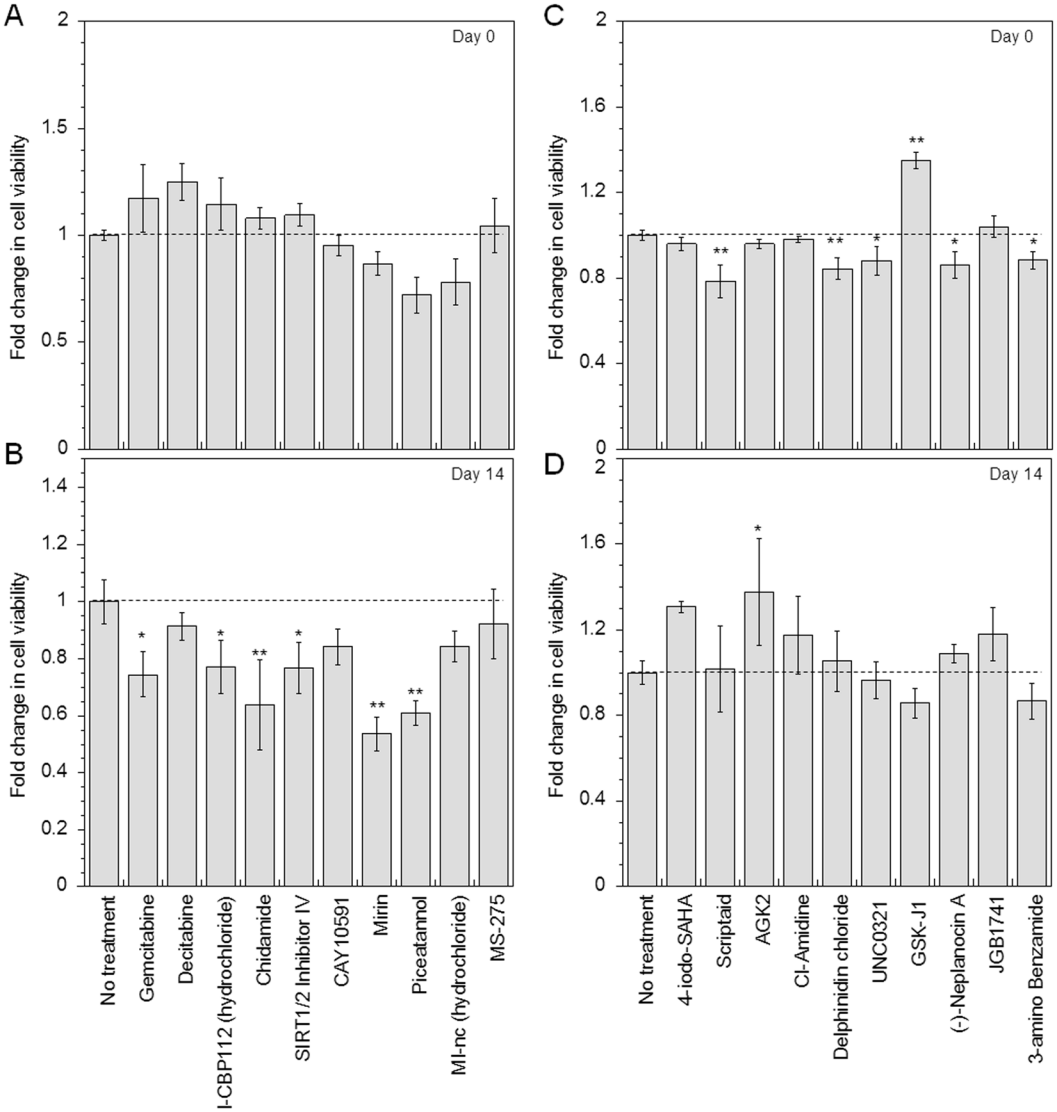
Effect of identified small molecule nucleosomal modifying drugs on cell viability. Cell viability was analyzed immediately post treatment with drugs (Day 0) and at 14 days post drug treatment (Day 14) using MTS cell viability assay. (**A**) and (**B**) show the effect on cell viability for drugs that increase osteogenesis, at Day 0 and 14, respectively. (**C**) and (**D**) show influence on cell viability for drugs that increase or decrease osteogenesis, at Day 0 and 14, respectively. Statistical analysis was performed using the Dunnett Multiple Comparisons test, which compares all columns versus a control column (no treatment). The symbols **and *represent a significant change in cell viability with respect to no treatment condition to the level of p < 0.01 and p < 0.05, respectively.

The effect of treatment with the library of pharmacologic agents on cell viability was assessed immediately post treatment (Day 0) and 14 days post treatment (Day 14) using MTS assay (Supplemental Fig. 2). Figure 3 shows the effect of the top 10 osteogenically sensitive drugs on the degree of cell viability at Day 0 and Day 14. Immediately post treatment (Day 0), >85% cell viability was maintained for all identified drugs, except *Piceatannol, MI-nc* and *Scriptaid*, which maintained at least 70% cell viability (Fig. 3A,C). On Day 14, cells that were pretreated with Decitabine, CAY10591, MI-nc and MS-275 maintained >80% cell viability (Fig. 3B). A significant decrease (at least p < 0.05) in cell viability was observed at 14 days with other compounds that increased osteogenesis including *Gemcitabine, I-CBP112, SIRT1/2 Inhibitor IV, Chidamide, Mirin* and *Piceatannol* (Fig. 3B). However, *Gemcitabine, I-CBP112* and *SIRT1/2 Inhibitor IV* maintained at least 70% cell viability and *Chidamide*, and *Piceatannol* maintained at least 60% cell viability. On the other hand, at 14-day post treatment no significant differences in cell viability compared to untreated cells were observed for small molecules identified for inhibiting osteogenesis (Fig. 3D).

### Specificity of identified compounds for osteogenesis versus adipogenesis

**Figure 4:**
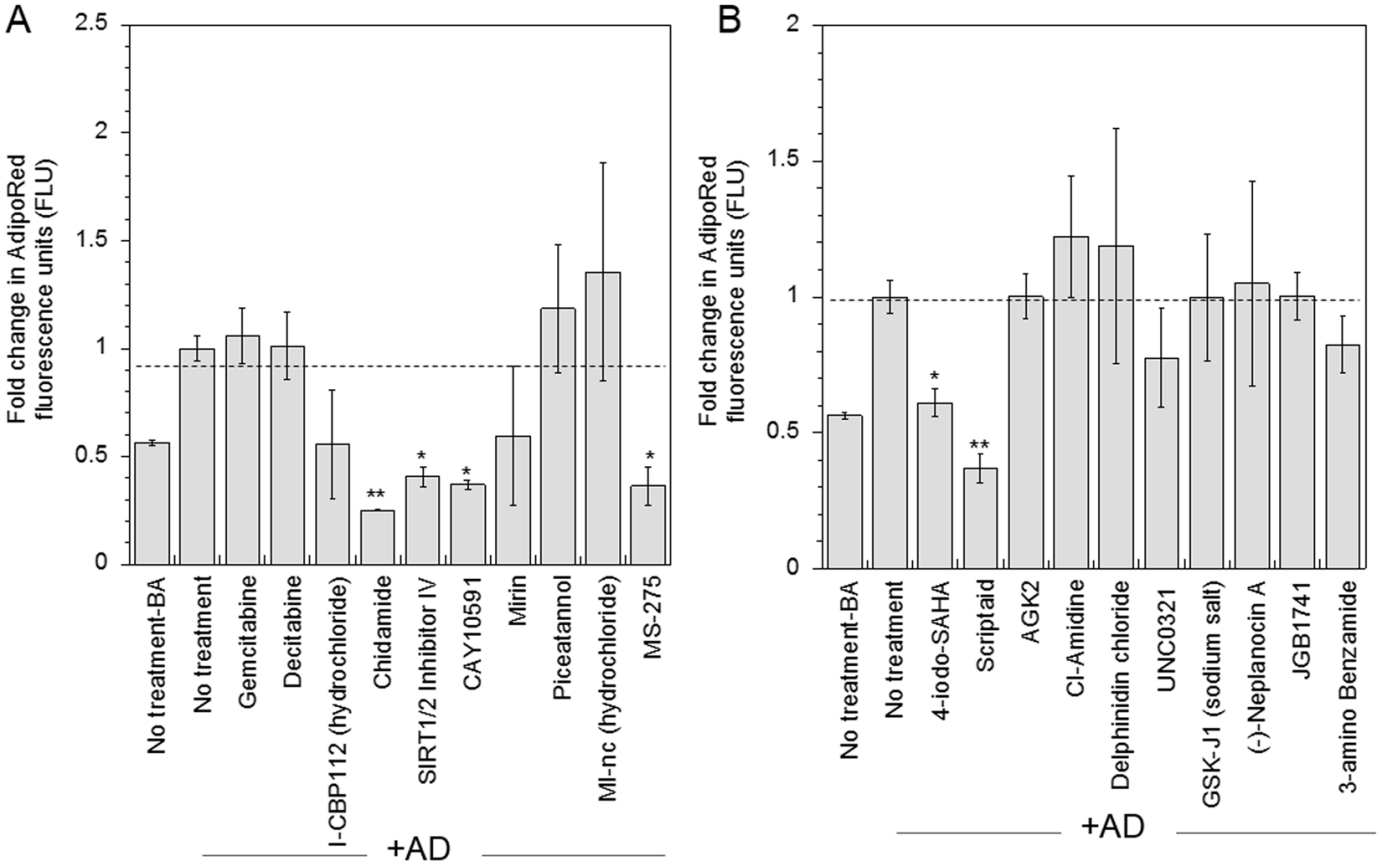
Effect of identified small molecule nucleosomal modifying drugs on adipogenesis. The specificity of identified drugs in influencing osteogenesis was evaluated by assessing the effect of drugs on adipogenesis. Cells treated with drugs identified to increase (**A**) and inhibit (**B**) osteogenesis were cultured in adipogenic differentiation media (AD) for 14 days and adipogenesis was evaluated using AdipoRed staining assay. Statistical analysis was performed using the Dunnett Multiple Comparisons test, which compares all columns versus a control column (AD-no treatment). The symbols ** and * represent a significant change in AdipoRed fluorescence units with respect to no treatment condition to the level of p < 0.01 and p < 0.05, respectively.

Next, we evaluated the specificity of the identified drugs in influencing the osteogenic versus adipogenic potential of hMSCs. The 10 drugs identified for significantly increasing osteogenesis did not similarly affect adipogenesis, and no increase in adipogenesis was observed. Interestingly, *Chidamide, SIRT1/2 inhibitor IV, CAY10591* and *MS-275* significantly decreased adipogenesis (at least p < 0.05) while *Gemcitabine, Decitabine, I-CBP112, Mirin, Piceatannol* and *MI-nc* did not significantly influence adipogenesis (Fig. 4A). Among the drugs identified for mediating inhibition of osteogenesis, *4-Iodo-SAHA* and *Scriptaid* significantly inhibited adipogenesis as well (at least p < 0.05), while *AGK2, CI-Amidine, Delphinidin Chloride, UNC0321, GSK-J1, Neplanocin A, JGB1741* and *3-Amino benzamide* did not significantly alter adipogenesis (Fig. 4B).

### Profiling change in nucleosomal organization post treatment with identified compounds using a high content image informatics platform

**Figure 5:**
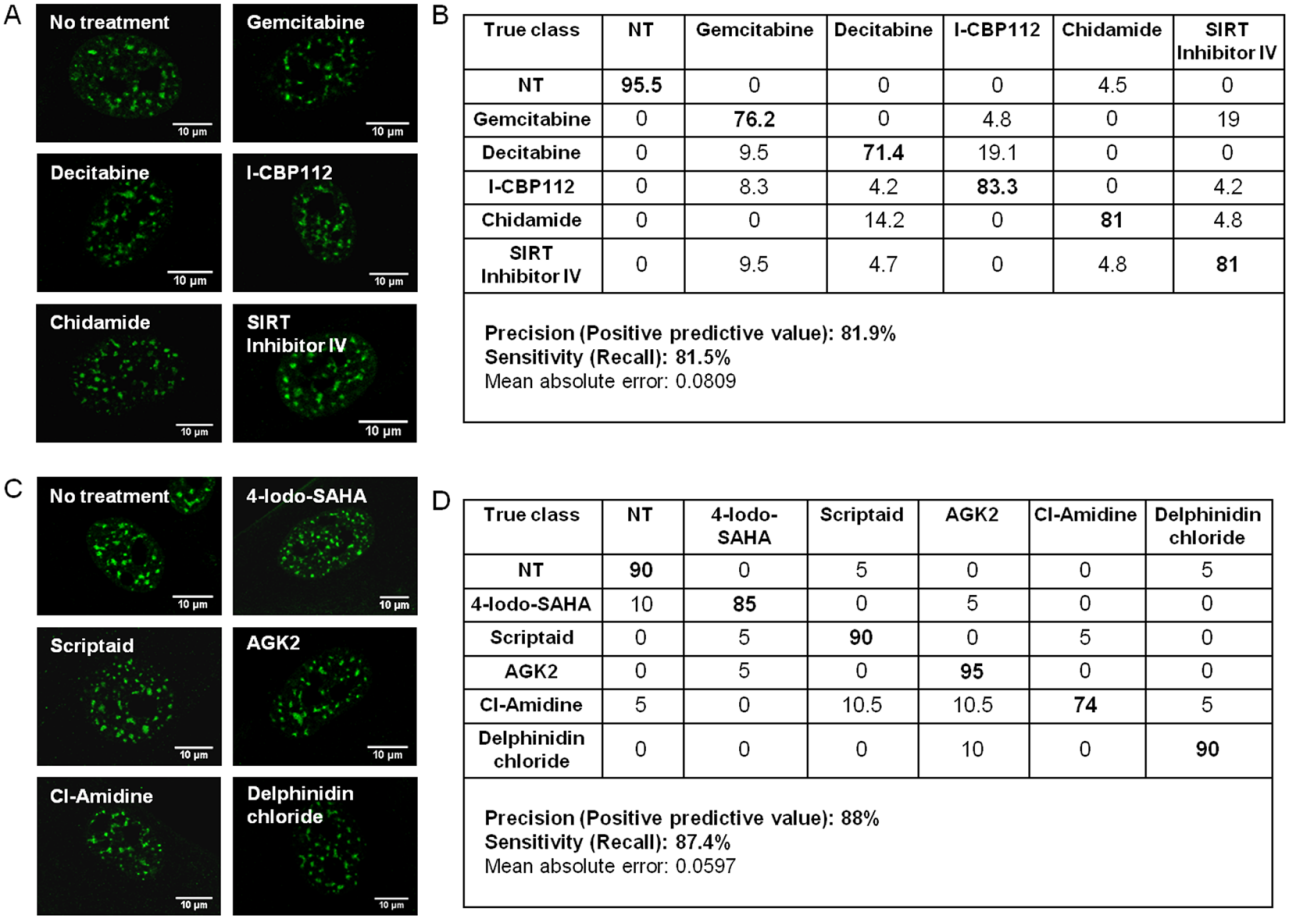
Using SC-35 organizational metrics, resultant epigenetic cell-states can be parsed immediately post treatment. (**A**) and (**B**) show representative SC-35 images taken immediately post treatment and classification table for small molecules that increase osteogenesis. (**C**) and (**D**) show representative SC-35 images and classification table for small molecules that inhibit osteogenesis.

Previously, we demonstrated that speckle factor SC-35 can be used as a surrogate marker to classify cell-state in response to external cues during osteogenic differentiation^20,23^. Our next step was to test if the high content image informatics approach could be employed to profile and elucidate the influence of the drugs on nucleosomal organization globally using SC-35 organizational metrics. We applied this platform to track changes in nucleosomal organization induced via top 5 key drugs identified to either enhance or inhibit differentiation (Fig. 2A,B). By employing high content analysis to compute organizational metrics of speckle factor SC-35 and using J48 decision tree classification, untreated cells and cells treated with drugs identified to enhance osteogenesis, namely, *Gemcitabine, Decitabine, I-CBP112, Chidamide* and *SIRT1/2 inhibitor IV*, could be parsed with >80% precision and sensitivity, immediately post treatment (Fig. 5A,B). Likewise, untreated cells and cells treated with drugs identified to inhibit osteogenesis, namely, *4-Iodo-SAHA, Scriptaid, AGK2, CI-amidine* and *Delphinidin chloride* could be parsed with > 85% precision and sensitivity based on variances in SC-35 spatial organization (Fig. 5C,D).

### Variations in nucleosomal organization post drug treatment and in presence of osteogenic cues can be profiled using SC-35 metrics within 72 hours

**Figure 6:**
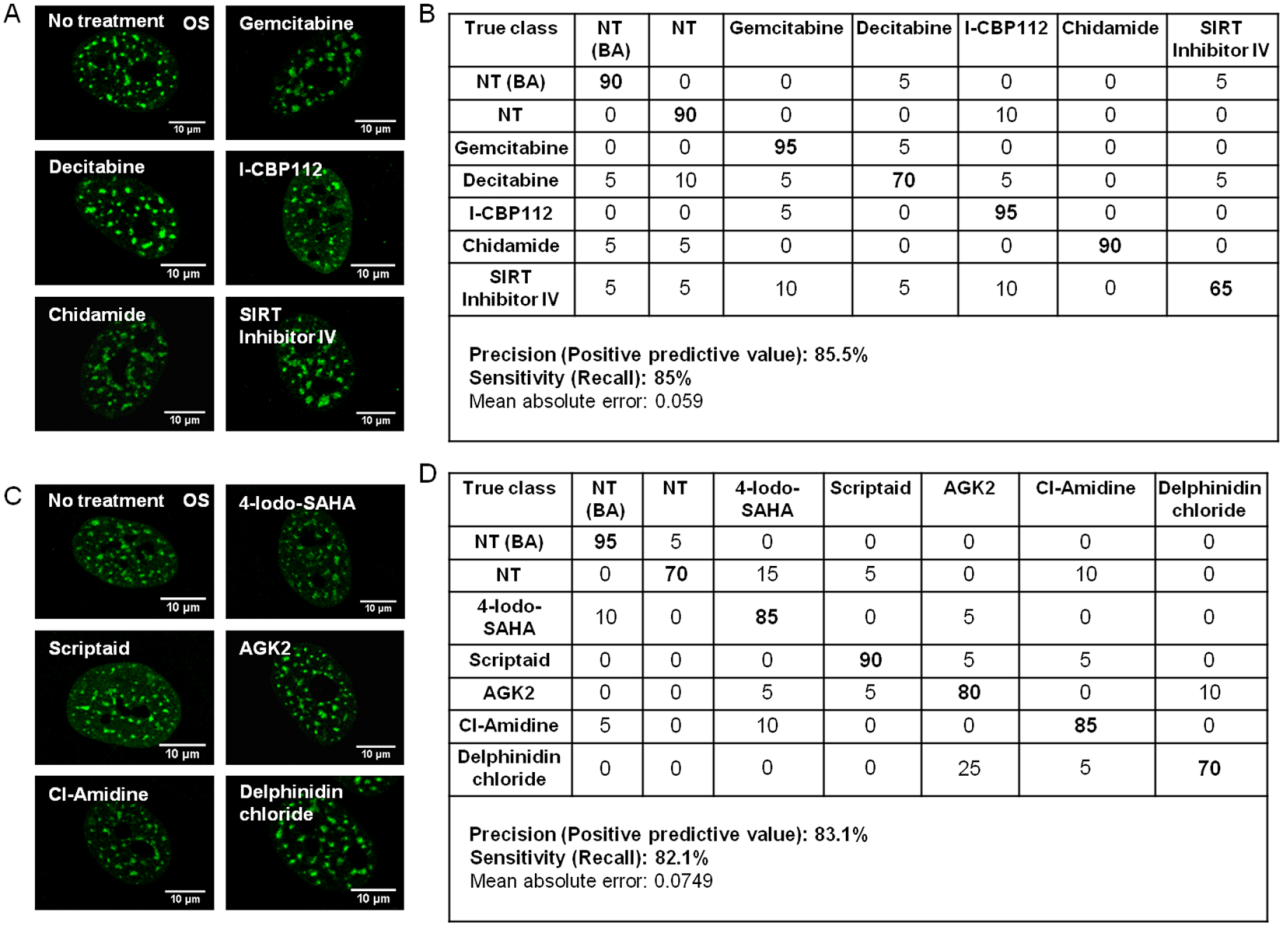
Image informatics of SC-35 nucleosomal organization can effectively parse the effect of epigenetic drugs on cellular phenotypes (cells were compared with and without drug pretreatment). (**A**) and (**C**) Representative SC-35 images of cells cultured in osteogenic medium (OS) for 3 days post treatment with various pharmacological agents identified to enhance and inhibit osteogenesis, respectively. (**B**) and (**D**) Classification table.

Next, we evaluated if cells pretreated with varied drugs and induced to differentiate using osteogenic cues could be profiled and parsed based on variations in nucleosomal organization present due to drug treatment. Our results indicate that using SC-35 organizational metrics, untreated cells and cells pre-treated with compounds capable of enhancing (Fig. 6A,B) or inhibiting osteogenesis (Fig. 6C,D) could be parsed with >80% precision and sensitivity in 72 hours post differentiation induction. Untreated cells or cells treated with pharmacological agents could also be differentiated from untreated cells cultured in basal condition with high fidelity (Fig. 6B,D). This indicates that the epigenetic modulations and lineage differentiation programs intersect within the nucleosome and can be forecast using SC35 organizational dynamics.

### Identified compounds enhance osteogenesis in hMSCs from an aged donor (>45 years) as well at a late passage number (>P7 hMSCs)

**Figure 7:**
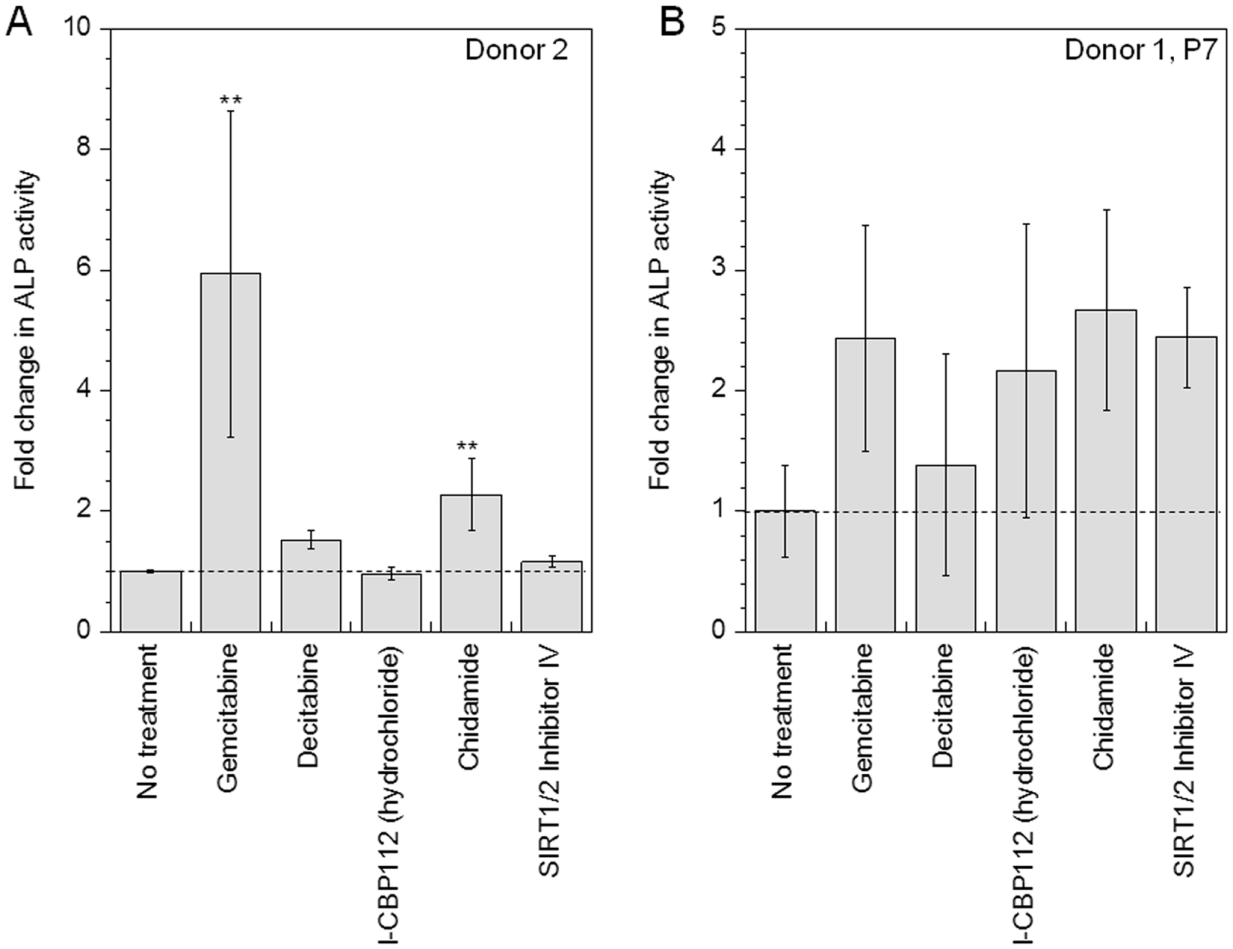
Identified drugs enhance osteogenesis in hMSCs from aged donor and at older passage number. (**A**) and (**B**) Influence of identified drugs on osteogenic differentiation was analyzed using ALP activity assay in cells from an older donor (age 46 years) and in cells at a late passage number (p7), respectively. Statistical analysis was performed using the Dunnett Multiple Comparisons test, which compares all columns versus a control column (no treatment). The symbol ** represent a significant change in ALP activity with respect to no treatment condition to the level of p < 0.01 and p < 0.05, respectively.

In order to test the hypothesis that epigenetic modifications can overcome regenerative tissue potential from aged donors, hMSCs derived from an aged donor (age: 46 years) were pre-treated with top 5 drugs identified to induce differentiation, namely *Gemcitabine, Decitabine, I-CBP112, Chidamide* and *SIRT1/2 Inhibitor IV*, and subsequently induced to differentiate. Osteogenic differentiation was assessed on Day 14 using ALP assay. It was observed, that even for cells from an aged donor, *Gemcitabine* and *Chidamide* significantly (p < 0.01) increased osteogenesis by 6- and 2.3- fold respectively, while *Decitabine* increased osteogenesis by 1.5-fold (Fig. 7A). These results indicate that these drugs effectively increase osteogenesis in hMSCs irrespective of donor age. On the other hand, *I-CBP112* and *SIRT Inhibitor IV* did not modulate differentiation in hMSCs from an older donor.

Next, we also evaluated if the identified drugs could enhance osteogenesis in cells at late passage number (P7). The identified drugs were observed to enhanced osteogenesis. (Fig. 7B). An increase in average ALP activity was observed for all five small molecules tested. However, statistical significant differences were not seen due to large error bars because of inherent variability and challenges faced in culturing hMSCs at a late passage number.

## Discussion

Transplantation of bone tissues is often needed to repair bones damaged due to severe trauma or disease^24,25^. Conventional autografts, allografts and xenografts have many associated adverse effects (implant rejection, disease transfer) and complications (cost, surgical risks and injury at donor site)^26,27^. Consequently, there is heightened demand for an alternative bone repair treatment using biocompatible and functional engineered-bone tissue formed from autologous stem cells. Mesenchymal stem cells (MSCs) are widely believed to be an ideal cell source to engineer bone tissue given their role in natural bone development, due to their availability and lineage-tunability^10,12,28^. However, the application of adult mesenchymal stem cells remains limited primarily due to decrease in cell’s developmental potential caused by *ex-vivo* culture and constraints of the advancing age of prospective donors.

**Figure 8:**
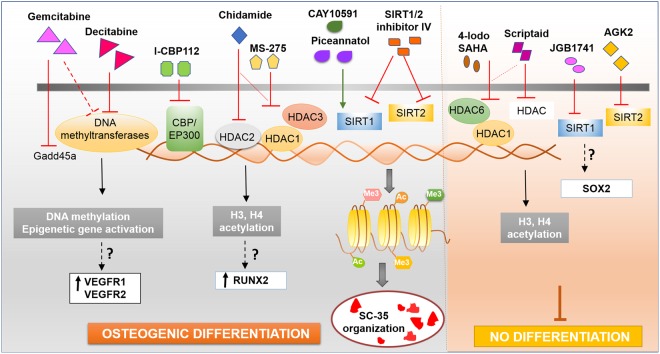
Small molecules that sensitively modulate osteogenic differentiation through epigenetic modifications. The different small molecules influence gene activity, chromatin remodeling and the spatial SC-35 domain organization.

While the role of epigenetics and nucleosomal modifications in cancer progression and treatment has been widely investigated^29^, the role of epigenetics in healthy stem cells and cellular differentiation remains relatively underexplored. Recently Attema *et al*. and Zardo *et al*. indicated that epigenetic mechanisms contribute to controlling stem cell potency and fate of hematopoietic stem cells^30,31^. Epigenetic modification such as DNA methylation and bivalent histone modifications have been observed to be associated in lineage-affiliated genes and lymphoid-affiliated genes, respectively^30^. Even so, the role of epigenetic programing in regulating the therapeutic potential of mesenchymal stem cell (MSCs) has not been elucidated. Our results demonstrated that epigenetic modifications regulate MSC differentiation potential and can be modulated using small molecule nucleosomal modifiers to direct optimal differentiation. In this study, we systematically studied 84 compounds and identified molecules capable of significantly influencing osteogenesis through epigenetic modifications. The key top hits identified (Fig. 8) to significantly and maximally increase or inhibit osteogenesis have been discussed in detail below.

Our study demonstrated that pre-treatment of cells with *Gemcitabine* and *Decitabine* significantly increased osteogenesis in hMSCs by 3.5-fold and 2.5-fold, respectively, while maintaining cell proliferation (growth). *Gemcitabine* and *Decitabine* are both nucleoside analogs of cytosine and share structural similarities. *Gemcitabine* specifically inhibits the Growth Arrest and DNA Damage inducible protein 45 a (Gadd45a), a key mediator of active DNA demethylation^32^, while *Decitabine* inhibits DNA methyltransferases and causes hypomethylation of cytosine residues in the absence of any significant mutagenic effect^33,34^. Both drugs have been used to treat various cancers. A recent study comparing the effects of *Gemcitabine* with *Decitabine* demonstrated that *Gemcitabine* functionally inhibits and destabilizes DNA methyltransferases and reactivates epigenetically silenced genes having activity equivalent to decitabine at concentrations significantly lower than those achieved in the treatment of patients with solid tumors^35^. Interestingly, decitabine and *Gemcitabine* have been shown to induce both VEGFR1 and VEGFR2 in A549 (adenocarcinoma) cells^35^. We believe that *Gemcitabine* and *Decitabine* enhance osteogenesis in bone marrow derived hMSCs possibly through induction of critical proteins such as VEGFR1 and VEGFR2 via epigenetic modulation, which regulate osteogenic differentiation of hMSCs^36,37^. Furthermore, the differential regulation of hMSC differentiation by *Gemcitabine* and decitabine can be a resultant of different mechanisms of actions and potency of the drugs, as decitabine alters DNA CpG methylation while *Gemcitabine* reactivates (induces) epigenetically silenced genes through an alternate mechanism.

Beyond *Gemcitabine* and *Decitabine*, *I-CBP112* was the next molecule identified to maximally increase osteogenesis. CBP (CREB (cAMP responsive element binding protein) binding protein (CREBBP)) and P300 (adenovirus E1A-associated 300 kDa protein) are two closely related histone acetyltransferases (HATs) and regulate gene-transcription. *I-CBP112* is a selective inhibitor of CBP and EP300 and directly binds their bromodomains that mediate their binding to acetylated lysine residues on histones and other proteins^38^. While CREB binding protein (CBP) and P300 were shown to play distinct roles in hematopoietic stem cell renewal and differentiation^39^, this is the first study to indicate their role in regulating MSC differentiation.

Many histone deacetylase inhibitors (HDIs) are among the small molecules identified in this study to epigenetically influence osteogenesis. HDIs are a new category of drugs that inhibit histone deacetylases (HDACs) and have been shown to influence cell growth, cycle, and differentiation. HDACs are further classified into classes depending on their location, for example, Class I HDACs are found in the nucleus and Class II HDACs alternate between the nucleus and cytoplasm. After *I-CBP112*, *Chidamid*e was the next molecule identified to maximally increase osteogenesis and is a benzamide HD1 that inhibits Class I HDAC1, HDAC2, HDAC3 as well as Class IIb HDAC10^40,41^. It inhibits epithelial-mesenchymal transition (EMT) in lung cancer cell lines and increases H3 acetylation levels^41–43^. *Chidamide*’s influence on osteogenic differentiation could possibly be through H3 acetylation based modifications, as H3 acetylation play a key role in maintaining the balance between genes associated with stem cell self-renewal and genes associated with osteogenic differentiation, for multipotent differentiation potential^44^, and increases the accessibility of osteocalcin promoter to the osteogenic transcription factors such as RUNX2. In addition to *Chidamide*, *MS-275* is another HDI identified to significantly increase osteogenesis. *MS-275* has been shown to preferentially inhibit HDAC1 over HDAC3, and does not inhibit HDAC8^45^. Franci *et al*. recently showed that treatment with *MS-275* modulated ESC fate by enhanced neural differentiation while preventing teratocarcinoma formation^46^. Our study demonstrates that priming MSCs with *MS-275* treatment also modulates their differentiation.

Small molecules maximally inhibiting osteogenesis were also identified in this study. Interestingly, two of the five small molecule drugs found to inhibit MSC differentiation are also classified as HDIs, namely *4-iodo-SAHA* and *Scriptaid*. *4-iodo-SAHA* is a hydrophobic derivative of the HDI *SAHA* (Suberoylanilide Hydroxamic Acid), and is a Class I and Class II inhibitor that affects the activity of both HDAC1 and HDAC6, like *SAHA*^47^. *SAHA* has been shown to induce cell apoptosis in several cancer cell lines and is being applied to cancer therapy^47–49^. Duncan *et al*. recently showed that *SAHA* induced apoptosis in dental pulp cells (DPCs) at high concentration (5 µM), while lower concentrations (1 µM), maintained cell viability and regulated pupal reparative events^50^. While *SAHA* (branded as Vorinostat) has been previously shown to enhance osteogenic differentiation of hMSCs^51^, our results indicate that pre-treatment of cells with *4-Iodo-SAHA* (1 µM), significantly inhibited both osteogenic and adipogenic differentiation, yet maintained cell viability. Similar results were observed for *Scriptaid*, which is also a HDAC inhibitor^52^ and has been studied in transformed cells^52,53^. Furthermore, we also tested *Trichostatin A*, a reversible inhibitor of class I, II, and IV histone deacetylases (HDACs) that has previously indicated in the likely enhancement of osteogenesis^18,54^. We observed that *Trichostatin A* did not influence hMCS differentiation (Supplemental Fig. 1). Taken together our results indicate that the varied class I and II HDAC inhibitors (*Chidamide, 4-iodo-SAHA, Scriptaid, Trichostatin A, MS-275*) have differential effect on hMSC differentiation as they have different mechanistic targets and are cell-type specific.

Another class of molecules that were among the top identified drugs was the *SIRT1* and *SIRT2* inhibitors and activators. *SIRT1* and *SIRT2* refers to sirtuin (silent mating type information regulation 2 homolog) 1 and 2 and are nicotinamide adenine dinucleotide (NAD)-dependent histone deacetylases (HDAC). They are considered Class III HDAC and differ from Class I and Class II as they require NAD^+^ for their deacetylase activity. SIRT1 and 2 have been shown to play a role in cancer^55,56^, aging^57^, differentiation^58,59^, and in regulating metabolic reprogramming and function of human pluripotent stem cells^60^. *SIRT1/2 inhibitor IV*, *CAY10591* and *Piceannatol* were among the top identified drugs to enhance osteogenesis and led to >2-fold increase in ALP activity. *SIRT1/2 inhibitor IV* is a cell-permeable inhibitor of SIRT1 and 2, and their NAD^+^-dependent deacetylase activity in a substrate competitive manner, without effecting class I and II histone deacetylases^61^, while CAY10591 and *Piceannatol* are known to activate SIRT1^62,63^. Recent studies have shown that SIRT1 influences multipotency of bone marrow derived stem cells by regulating SOX2 in the nucleus^64^, and activation of SIRT1 using resveratrol enhanced osteogenesis in cells^64,65^. Interestingly, our results indicate that combined inhibition of *SIRT1* and *2* using *SIRT1/2 inhibitor IV* significantly increased osteogenic differentiation (p < 0.01). Our results also show that activation of SIRT1 using *CAY10591*^62^ and *Piceannatol* significantly increased osteogenesis (at least p < 0.05). Interestingly, while *Piceannatol*, a resveratrol analog^66,67^, increased ALP activity, treatment with trans-resveratrol, a compound known to inhibit cyclooxygenase 1 (COX-1)^68^ and activate SIRT1^69^, did not influence osteogenesis in hMSCs (Supplemental Fig. 1). On the other hand, we also observed that specific inhibition of *SIRT1* using *JGB1741*^70^ and specific inhibition of SIRT2 (without effecting SIRT1 and 3), using *AGK2*^71^, decreased osteogenesis without influencing adipogenesis or decreasing cell viability.

After identifying small molecules that influence osteogenic differentiation of hMCSs *in-vitro* through epigenetic modifications, we tested the effect of these molecules in mediating osteogenesis in aged hMSCs. Our results showed that *Gemcitabine* and *Chidamide* maximally increased osteogenic differentiation in hMSCs obtained from aged donors by 5.9- and 2.3- fold, respectively, and in hMSCs at late passage number by 2.4- and 2.6- fold respectively. Frazen *et al*. recently showed that epigenetic modifications are associated with senescence in hMSCs^72^. Our study has further demonstrated that age related loss of differentiation potential can be rescued through modulating epigenetic modifications by innovatively employing small molecules.

Current technologies like chromatin immunoprecipitation methods (e.g., CHIP) to elucidate the nucleosomal alterations in histone modifications are based on population averaged metrics. These methods are unable to catalog minute epigenetic variations in single cells that regulate the heterogeneity of cell-fate decisions^73,74^. These methods are also unable to profile the changes in *in-situ* nucleosomal organization elicited due to treatment by small molecules. Previously we have demonstrated that organizational metrics of speckle factor SC-35 are sensitive to signaling molecules mediating osteogenic differentiation in response to external cues and can be employed to parse emergent MSC phenotypes, within 72 hours of exposure^20,23^. Further, during hMSC differentiation, SC-35 domains co-localize with loci of active gene transcripts in the nucleus leading to increased expression^23^. SC-35 domain organization is therefore dynamic and regulated by nucleosomal organizational changes and gene transcription. In this study, our results demonstrate that exposure to the various small molecules produces distinct nucleosomal organizational profiles and that speckle factor SC-35 can also be employed as a surrogate marker to profile these *in-situ* variances in the nucleosomal organization. Using SC-35 organizational metrics and machine learning approaches, cells primed with different small molecules could be parsed with a precision of >85%. Furthermore, our results also indicate that SC-35 organization is highly sensitive (>85%) and modulated by underlying epigenetic modifications as they influence chromatic modeling and gene activation.

In summary, this study demonstrates that small molecules can be applied to modulate stem cell fate and direct cell development. In this study we have successfully identified novel indications for key small molecules to direct cell differentiation of hMSCs through epigenetic modulation. We have further shown that specific identified molecules also increase cellular differentiation in hMSCs obtained from an aged donor as well as in hMSCs at a late passage. Our results elucidate that the underlying epigenetic profile is a pivotal regulator of MSC potential and could afford an approach to design adult stem cell-based tissue engineering strategies. Further studies need to be conducted to decipher the mechanistic role of identified molecules in MSCs and develop strategies for implementing their applications in tissue engineering.

## Methods

### Cell culture

Human mesenchymal stem cells (hMSCs) were obtained from Texas A&M University (College Station, TX). Cells were cultured in a humidity-controlled environment under 5% CO_2_ and 37 °C and fed every 3 to 4 days with basal growth media (BA) consisting of Alpha Minimum Essential medium (αMEM) with L-glutamine (Life Technologies) supplemented with fetal bovine serum (10% v/v, Atlanta Biologicals) and penicillin-streptomycin (0.1% v/v, Life Technologies). Cells were received at passage 1 and used for up to 5 passages, for both young and old donors, unless specified otherwise as per experimental conditions. Osteogenic differentiation (OS) was induced by culturing hMSCs in BA media supplemented with 0.5 mM L-ascorbic acid-2-phosphate, 0.2 µM dexamethasone (dex), and 20 mM β-glycerophosphate. Adipogenic differentiation (AD) was induced with BA media supplemented with 1 µM dexamethasone, 50 µM indomethacin, 10 µg/ml insulin, and 100 µM 3-isobutyl-1-methyl-xanthine. Cells were allowed to adhere overnight in basal growth media, followed by a media change with appropriate induction media. All culture reagents were purchased from Sigma-Aldrich unless otherwise specified.

### Treatment with small molecule pharmacological agents

An epigenetic screening library of small molecules (Cayman Chemicals) was utilized and 84 compounds from the library were screened to determine the molecules capable of influencing osteogenesis. The library included compounds that modulate the activity of methyltransferases, demethylases, HATs, HDACs and acetylated lysine reader proteins. To evaluate the effect of the small molecules, cells were seeded in basal medium overnight, and subsequently treated with pharmacological agents for 24 hours at concentrations recommended by the manufacturer. 24 hours post drug treatment, the medium was replaced with fresh medium of differentiation medium as per experimental conditions.

### Immuno-fluorescence staining

hMSCs were seeded on eight-chamber glass slides (Nunc, Rochester, NY) at a density of 10,000 cells/cm^2^ and cultured in various conditions as required by the experiment. At desired time point for analysis, cells were fixed in 4% paraformaldehyde in PBS for 15 min, permeabilized with 0.1% Triton-X in PBS for 5 minutes, and blocked by incubating in blocking buffer (5% NGS and 1% BSA in PBS) for 1 hour at room temperature. For performing immunostaining for SC-35, samples were first incubated overnight with primary antibody (Abcam) in blocking buffer at a 1:500 ratio, followed by three 10-minute washes in blocking buffer. Next, samples were incubated with secondary antibody solution (Alexa Fluor; Invitrogen) in blocking buffer at a 1:250 ratio for 1–1.5 hours at room temperature, followed by three 10-minute washes with blocking buffer. All samples were counterstained with 5 μg/ml Hoechst (Sigma) in PBS and stored at 4 °C until imaging.

### Differentiation assays

hMSCs were cultured for 14 days in differentiation induction medium prior to assessing differentiation. To analyze adipogenic differentiation, Adipo Red staining assay for staining intracellular triglycerides was performed on fixed cells as per manufacturer’s protocol (Lonza). For analyzing osteogenic differentiation, cells were either fixed or lysed at day 14. Alkaline phosphatase (ALP) was assessed by staining fixed cells using Fast Blue RR (Sigma) or by performing calorimetric assay ALP assay (Biovision Inc) on cell lysate as per manufacturer’s protocol. DNA content in cell lysate was determined by performing the PicoGreen assay (Life Technologies) as per manufacturer’s protocol. Analysis of ALP activity using calorimetric ALP assay was performed and represented after normalization by DNA content.

### Assessing cell proliferation

The CellTiter 96® AQueous One Solution Cell Proliferation Assay (Promega) was performed to determine the cytotoxicity and proliferation of cells as per manufacturer’s protocol. Briefly, cells were cultured at a density of 10,000 cells/cm^2^ in a 96 well plate and treated with pharmacological agents. After treatment the media was replaced with fresh basal medium and cells were maintained in basal medium for up to 14 days. To assess cell viability, immediately post treatment and 14 days after treatment, Aqueous One Solution was added in each well to be assayed and incubated for 1 hour. The fluorescence was measured using a plate reader at 490 nm.

### High content image analysis of nuclear speckle factor (SC-35) Organization

High content image analysis for SC-35 organization was performed using the similar methodology to the one described in Vega, S *et al*.^20^. Briefly, high resolution 1024 × 1024 images were first acquired for cells stained for speckle factor SC-35 using antibody staining and for DNA using Hoechst dye staining (as described above.), using 63x and 1.3 NA objective with a Leica TCS SP8 system (Leica Microsystems). To analyze the organization of SC-35, 26 texture-based Haralick texture features (13 mean and 13 standard deviation or range descriptors) were subsequently acquired for each cell using high content image analysis. First, images underwent intensity-based thresholding to create nuclear ROI masks for each nucleus in a given image, based on Hoechst DNA staining. Next, Haralick descriptors were obtained using a Matlab algorithm. A complete list of the calculated Haralick descriptors with their definitions is provided in Table S1. These descriptors are quantifiable measurements of texture features that represent the spatial organization of the SC-35 in the nucleus. The 26 descriptors were linearly reduced to a minimum number of eigenvectors that account for 95% variance of the data by performing principal component analysis (PCA) using the Weka (Waikato Environment for Knowledge Analysis) open source software (University of Waikato, New Zealand).

To illustrate differences between the various subpopulations, a predictive classification model was made using J48 decision tree analysis in the Weka software. J48 generated a C4.5 pruned decision tree, where tree pruning is used as a tool to correct for potential over fitting. The best performing classification tree was generated by using the experimental data as the training set. The quality of the tree is reported in terms of the percent of correctly classified instances, precision (positive predictive value), and recall (sensitivity). Briefly, Precision = TP/(TP + FP) and Recall = TP/(TP + FN). True positives (TP) are the number of instances correctly classified as belonging to the positive class. False positives (FP) are the number of instances incorrectly classified to the class. False negatives (FN) are the number of instances not classified to the class but belong to class. Precision is also defined as the number of instances that truly have class *x* among all those which are classified as class *x*.

### Statistics

All statistical analysis was performed using the computer program Instat (GraphPad, San Diego, CA). Experiments were statistically analyzed using the Tukey-Kramer Multiple Comparisons test, which compares all pairs of columns, using a 95% confidence interval or using the Dunnett Multiple Comparisons test which compares all columns versus a control column.

## Electronic supplementary material

Supplementary Information

## Data Availability

The datasets generated during and/or analyzed in this study are available from the corresponding author on reasonable request.
