# Supplementary material for: Engineering Lineage Potency and Plasticity of Stem Cells using Epigenetic Molecules
Source: Sci Rep. 2018 Nov 2;8:16289. doi: 10.1038/s41598-018-34511-7 (PMC6215020; doi:10.1038/s41598-018-34511-7)
Supplement: Supplementary file 1 — Supplementary Information [file 41598_2018_34511_MOESM1_ESM.pdf]

Engineering Lineage Potency and Plasticity of Stem Cells using Epigenetic Molecules

Anandika Dhaliwal[1], Sandra Pelka[1], David Gray[1] and Prabhas V. Moghe[1,2*]

[1]Department of Biomedical Engineering, Rutgers University, Piscataway, NJ
[2]Department of Chemical and Biochemical Engineering, Rutgers University, Piscataway, NJ
*moghe@rutgers.edu

SUPPLEMENTAL INFORMATION

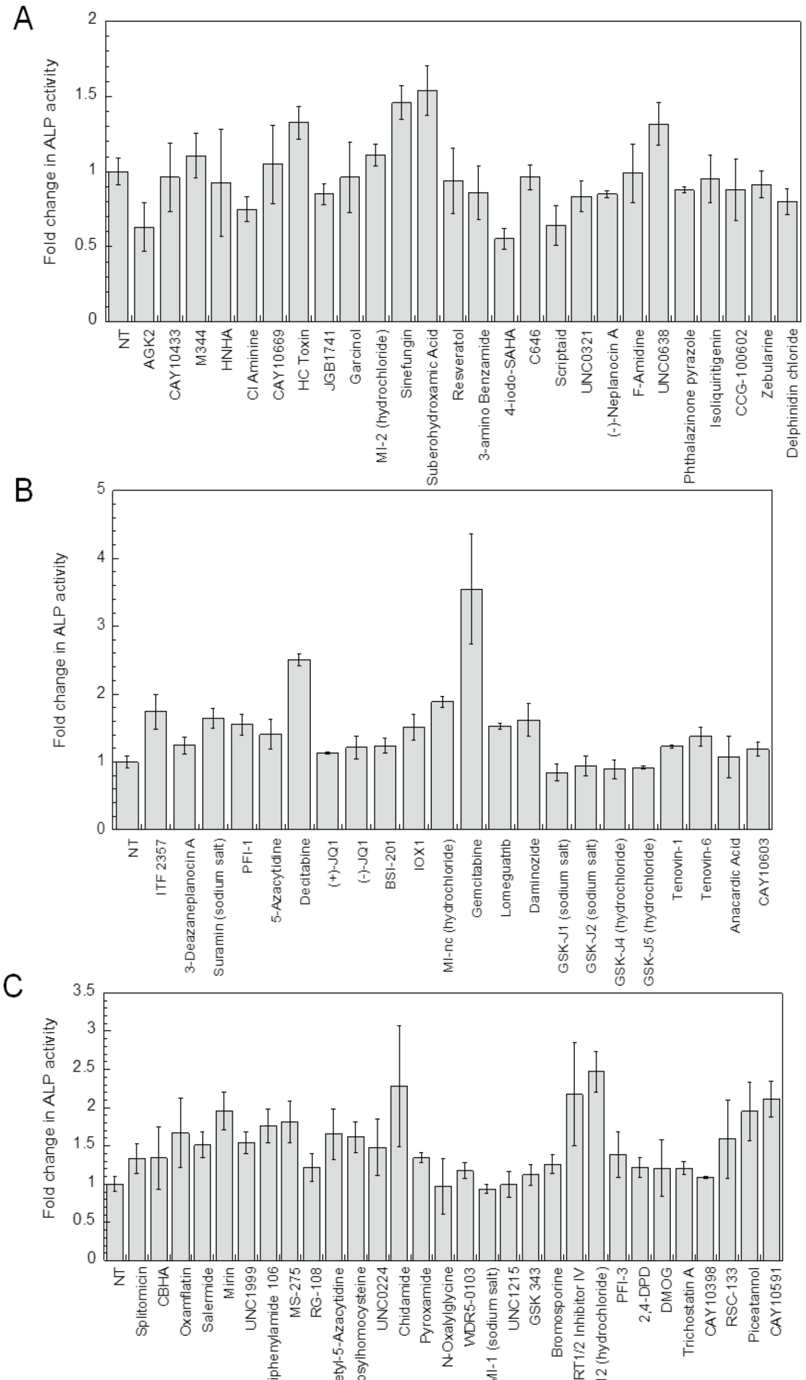

Supplemental Figure 1: Effect of drug treatment of osteogenic differentiation. hMSCs were cultured in presence of osteogenic medium for 14 days post treatment with small molecule drugs and osteogenic differentiation was assessed using ALP calorimetric assay. ALP activity was normalized to total DNA content, and fold change with respect to no-treatment was plotted. **A)** Data for drug 1- drug 29, **B)** Data for drug 30-53, and **C)** Data for drug 54-84.

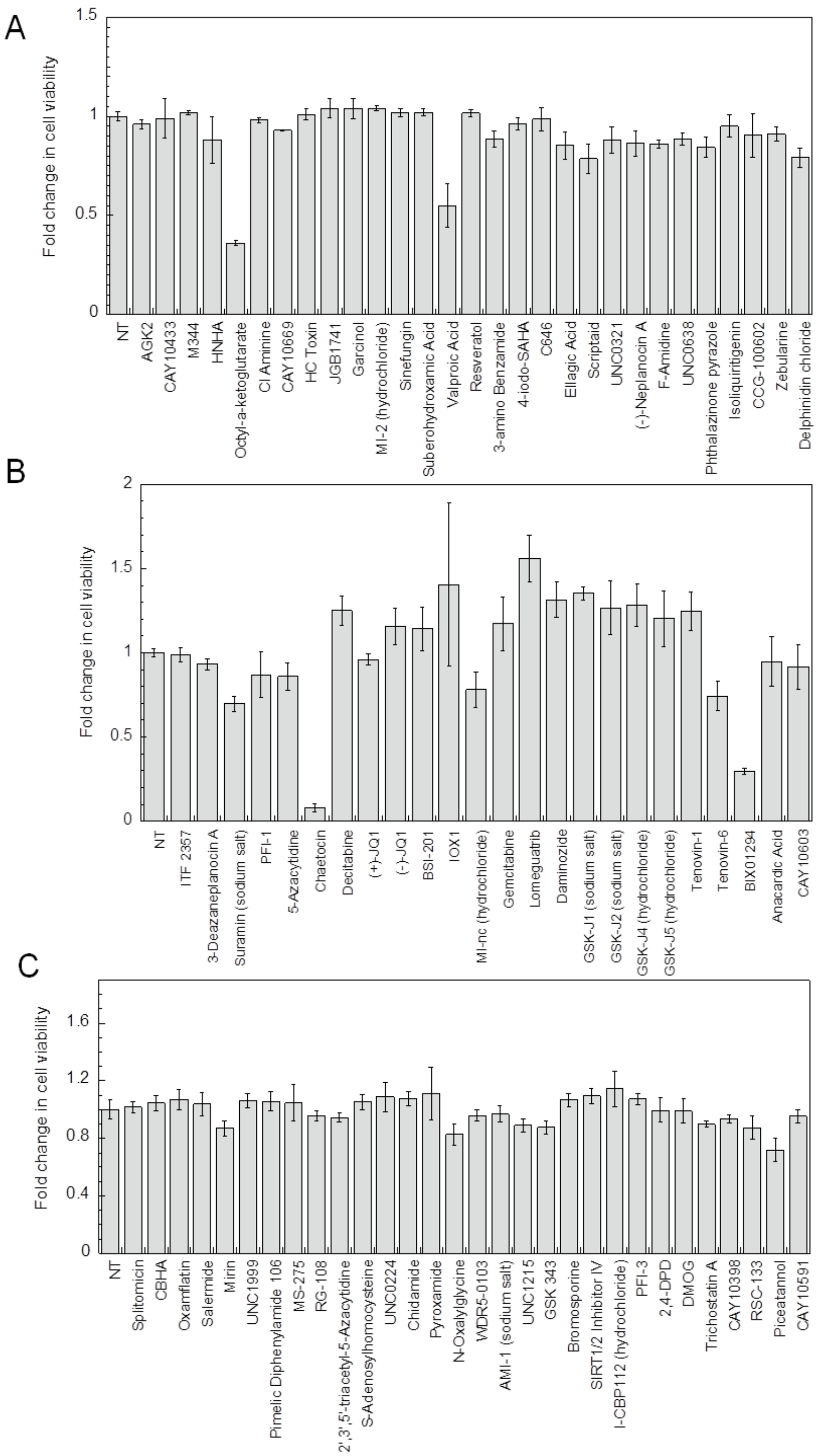

Supplemental Figure 2: Effect of drug treatment of cell viability, assessed immediately after drug treatment (Day 0) using MTS cell viability assay. A) Data for drug 1- drug 29, **B)** Data for drug 30-53, and **C)** Data for drug 54-84.

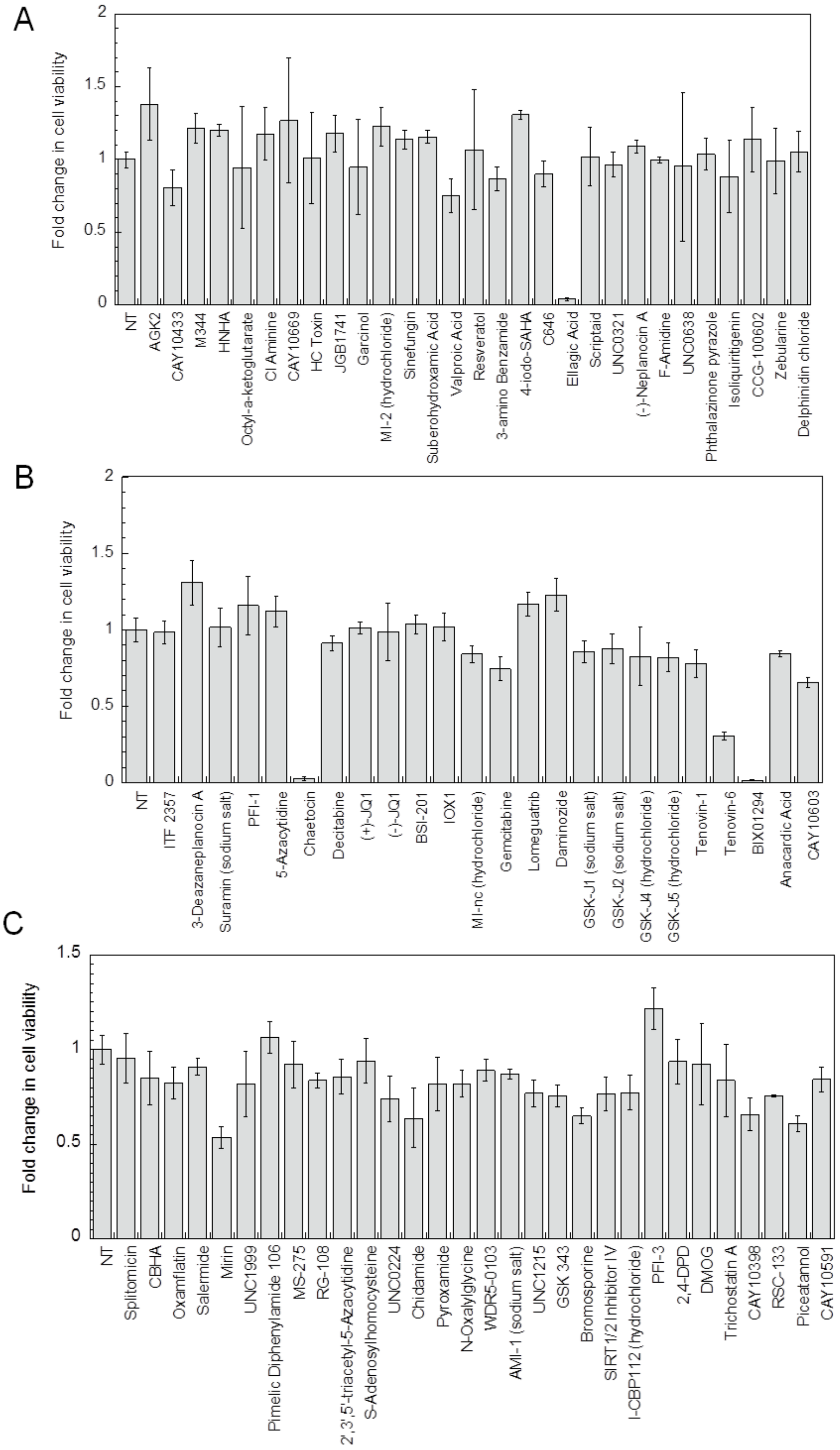

Supplemental Figure 3: Effect of drug treatment of cell viability, assessed 14 days (Day 14) after the treatment using MTS cell viability assay. **A)** Data for drug 1- drug 29, **B)** Data for drug 30-53, and **C)** Data for drug 54-84.

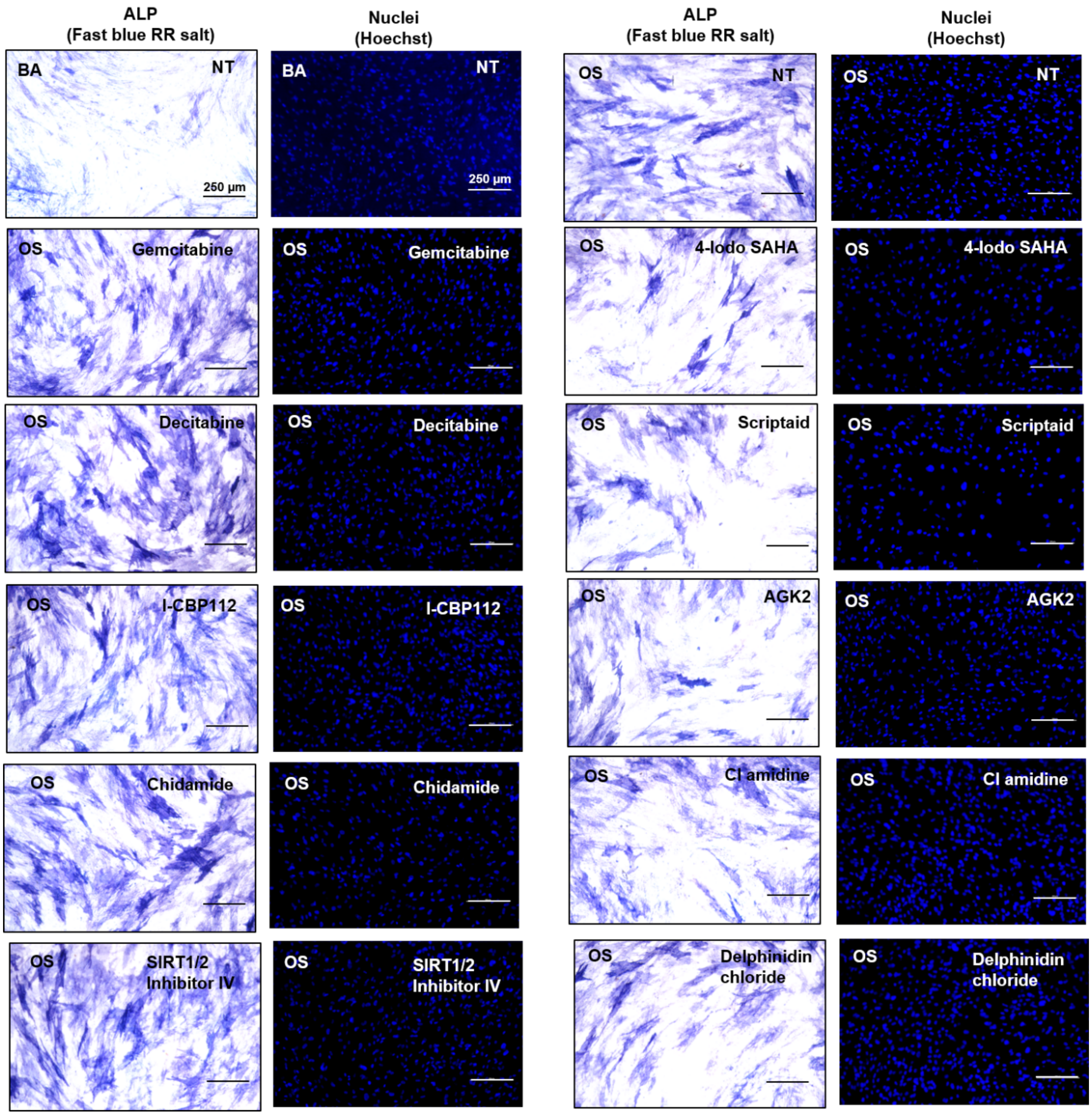

Supplemental Figure 4: Effect of drug treatment on osteogenic differentiation, assessed at 14 days using Fast Blue staining.

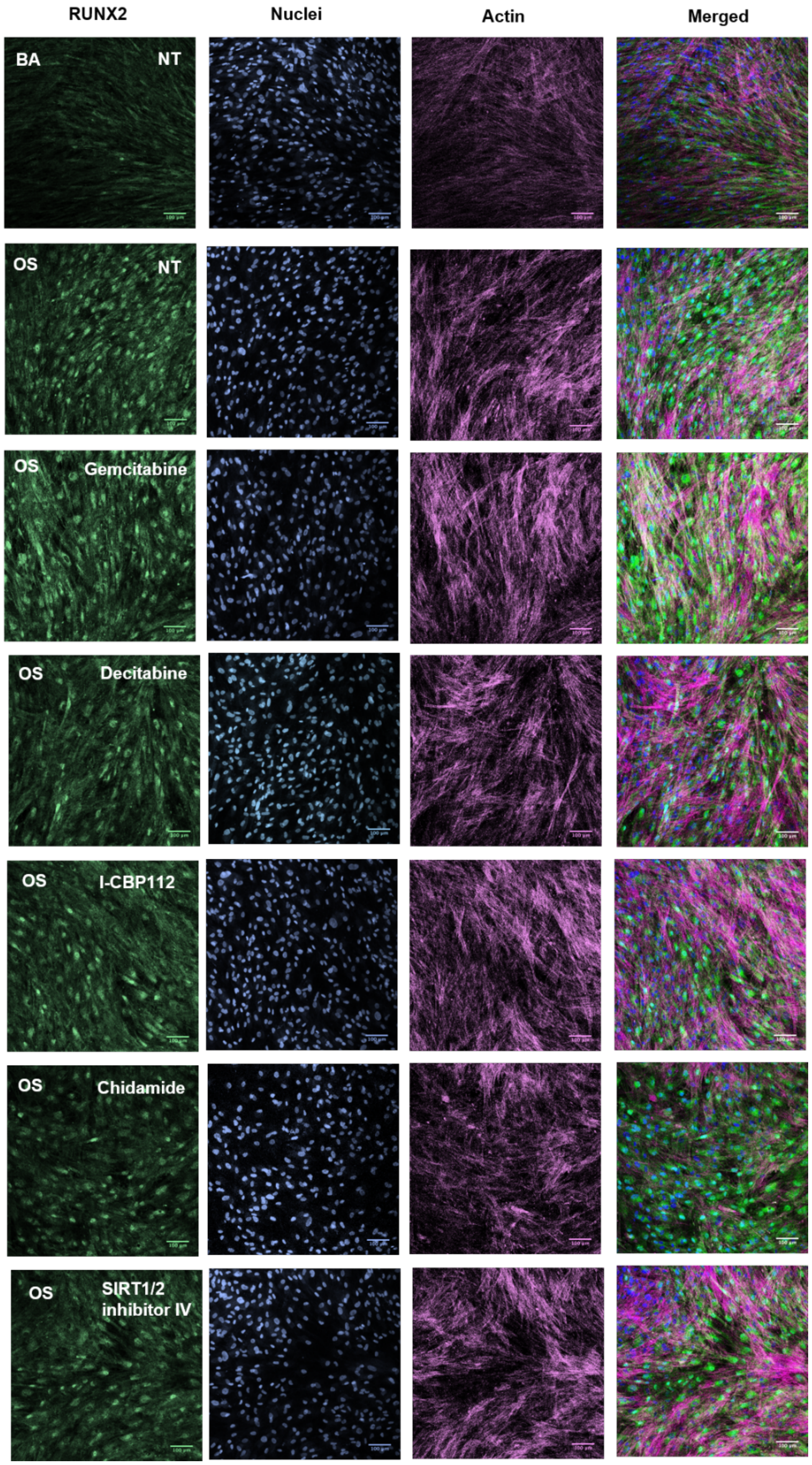

Supplemental Figure 5: Effect of treatment with select identified drugs on cell morphology and osteogenic differentiation, assessed through staining for actin using phalloidin staining, nuclei using hoechst dye staining and osteogenic marker RUNX2 using immune-staining.

Supplemental Table 1: List of the top 10 small molecule drugs that most increase osteogenesis. The concentrations used for treatment, chemical structures and primary mechanism of action have been listed.

Drug	Dose	Structure	Mechanism
Gemcitabine	0.07 µM	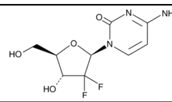	Gadd45a inhibitor
Decitabine	2.5 µM	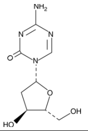	DNA methyltransferase inhibitor
I-CBP112	0.3 µM	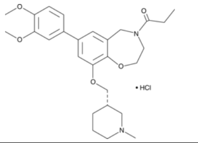	CBP/EP300 inhibitor
Chidamide	4 µM	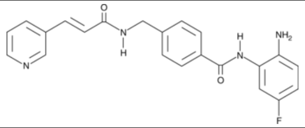	HDAC inhibitor
SIRT1/2 Inhibitor IV	50 µM	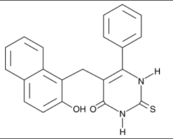	SIRT1, SIRT2 inhibitor
CAY10591	40 µM	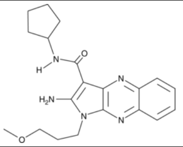	Activates SIRT1
Mirin	60 µM	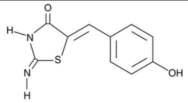	Inhibits MRN-dependent phosphorylation of H2AX
Piceatannol	30 µM	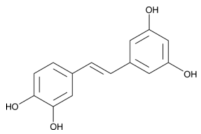	Resveratrol analog, induces SIRT1,<br>Also inhibits tyrosine and serine/threonine protein kinases
MI-nc (hydrochloride)	200 µM	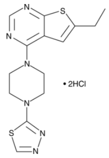	Inhibits menin-MLL interaction
MS-275	4 µM	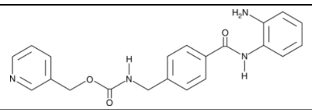	HDAC inhibitor

Supplemental Table 2: List of the top 10 small molecule drugs that most inhibit osteogenesis. The concentrations used for treatment, chemical structures and primary mechanism of action have been listed.

Drug	Dose	Structure	Mechanism
4- Iodo-SAHA	1 µM	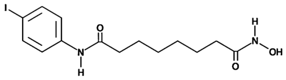	HDAC1, HDAC6 inhibitor
Scriptaid	7 µM	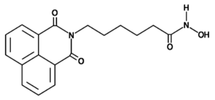	HDAC inhibitor
AGK2	3.5 µM	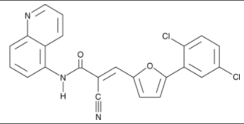	SIRT2 inhibitor
Cl-Amidine	6 µM	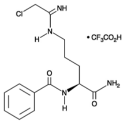	PAD4 inhibitor
Delphinidin chloride	20 µM	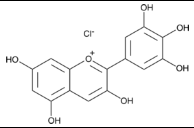	Histone acetyltransferase (HAT) inhibitor
UNC0321	0.01 µM	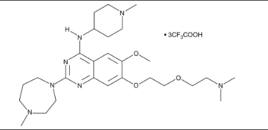	Inhibits HMTase G9a
GSK-J1	0.05 µM	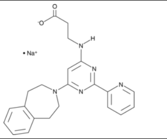	Inhibits H3K27 histone demethylases JMJD3 and UTX
(-)-Neplanocin A	0.5 µM	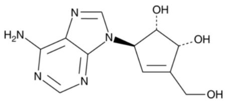	Inactivates SAH hydrolase; Indirectly inhibits SAM-dependent methyltransferase
JGB1741	15 µM	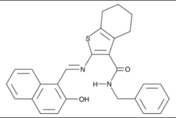	Inhibits SIRT1
3-amino Benzamide	50 µM	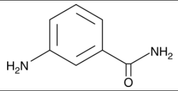	Inhibits poly(ADP-ribose) polymerases
